# Defining preconception: exploring the concept of a preconception population

**DOI:** 10.1186/s12884-020-02973-1

**Published:** 2020-05-07

**Authors:** Briony Hill, Jennifer Hall, Helen Skouteris, Sinéad Currie

**Affiliations:** 1 Monash Centre for Health Research and Implementation, School of Public Health and Preventive Medicine, Monash University, Level 1, 43-51 Kanooka Grove, Clayton, Victoria, 3168 Australia; 2 EGA Institute for Women’s Health, University College London, 74 Huntley St, London, WC1E 6AU UK; 3 Psychology, Faculty of Natural Sciences, University of Stirling, Stirling, FK9 4LA UK

**Keywords:** Preconception, Pregnancy intention, Pregnancy planning, Public health

## Abstract

**Background:**

Health prior to conception can significantly impact offspring health, however, a clear definition of the attributes of the preconception population is currently lacking. We aimed to use existing literature to explore the concept and attributes of a preconception population by: [1] identifying characteristics and research recruitment methods; and [2] generating an attribute-based working definition of a preconception population.

**Methods:**

A rapid review of current literature using CINAHL and the subject heading ‘pre-pregnancy care’ was conducted (Stage 1). Data extracted included definitions of preconception, participant inclusion/exclusion criteria, participant characteristics, and recruitment methods. Stage 2 involved a wider search of relevant publications beyond peer-reviewed literature followed by a concept analysis of the phrase “preconception population” applying Walker and Avant’s framework (Stage 2).

**Results:**

Twenty-three papers (19 studies) were included in Stage 1. “Preconception” was explicitly defined in one study. Twelve studies specified participants must be planning a pregnancy. Stage 2 included 33 publications. Four key perspectives for the concept of the preconception population were derived: [1] intentional; [2] potential; [3] public health; and [4] life course.

**Conclusions:**

Adopting these perspectives may allow researchers to accurately define, identify and recruit preconception populations and to develop interventions that are appropriately broad or tailored depending on population needs. We hope the definitions will facilitate research with this population and will subsequently improve the wellbeing of preconception men and women, which is essential to ensuring the health of future generations.

## Background

Preconception health is critically important to promote favourable maternal and infant outcomes in both the short- and long-term. Adverse lifestyle factors and unhealthy weight status prior to pregnancy are now recognised as important factors associated with reduced fertility [1], excessive gestational weight gain [2], postpartum weight retention, and high long-term weight status in both mothers and offspring [3]. Furthermore, the preconception period is considered a unique opportunity for the reduction of risk factors linked with non-communicable diseases in offspring [4]. Mechanisms by which maternal preconception health can impact offspring health include epigenetic alterations to gene expression occurring soon after conception [5], via poorer pregnancy outcomes associated with maternal over- or undernutrition [5], or via environmental and social impacts [6].

In 2006, the Centers for Disease Control and Prevention (CDC) published a report of recommendations to improve preconception health and care in the US [7]. Since then, there has been a marked increase in preconception research and international and national health bodies [2, 8, 9], including the World Health Organization [10], have identified preconception as a key life phase for health promotion.

However, there is a lack of consistency in the use of the term “preconception” leading authors to preach an absence of useful definitions [11], in particular with regard to defining the preconception population. This is despite the fact that concepts relevant to preconception populations have been outlined in detail. Yet these concepts focus on the “what” and the “how”, for example improving the health of reproductive age women (“what”) and reproductive life planning (“how”), rather than focusing on the “who” i.e. defining the key attributes of a preconception population [12, 13]. Understanding the *who* is essential for the progression of appropriate research and designing targeted interventions to promote health preconception. Indeed, in research, defining your population is a methodological requirement for selecting your study sample [14].

With this issue in mind, the 2018 *Lancet* series on preconception health posited three perspectives upon which to conceptualise the preconception period [15]. These were: [1] the biological perspective, encompassing the days to weeks before embryo development [2]; the individual perspective, which includes a conscious intention to conceive, and is typically weeks to months before pregnancy; and [3] the public health perspective, which occurs over the longer period of months to years with the goal to address preconception risk factors. These definitions provide a useful, evidence-based framework to identify critical preconception time points, as well as upon which to target various individual and public health initiatives to improve preconception health outcomes. However, they do not go so far as to identify the specific attributes of potential preconception populations.

With the absence of an agreed definition of ‘preconception’, researchers, health professionals, and policy makers have had to form their own, leading to confusion and a lack of comparability. Given the importance of preconception health for future maternal and offspring health, our understanding of preconception populations must deepen. Robust and appropriately targeted research are dependent on understanding the characteristics of the group, yet no studies have specifically investigated the characteristics or attributes of “preconception” that will enable the population to be defined and targeted effectively. To this end, the overall aim of this paper was to explore the concept and attributes of the preconception population. Specifically, we aimed to [1] identify the characteristics of preconception populations in the literature and identify recruitment methods of this group; and [2] use a concept analysis framework [16] to generate an attribute-based working definition of a preconception population informed by existing literature.

## Methods

### Stage 1: identifying common characteristics of preconception populations

Stage 1 addressed the first aim of this study, to identify the characteristics of preconception populations in peer-reviewed literature and explore the recruitment methods of this group. Recruitment methods were specifically explored because they centre on the target population, which was the concept of interest for this study.

#### Search strategy

A rapid literature search was performed using the Cumulative Index to Nursing and Allied Health Literature (CINAHL) Complete database. The subject heading ‘pre-pregnancy care’ was used to identify relevant articles that included related terms such as preconception care, pre-conceptual, attempting conception, preconception, and peri-conceptual. The search was limited to articles published within the last 10 years to focus on a contemporary definition of preconception populations.

#### Eligibility criteria

Studies were required to: [1] recruit participants (men and/or women) for primary research during the preconception period (not pregnant at recruitment) [2]; refer to the population as preconception (or related term e.g. pregnancy planning) in the title or abstract [3]; provide clear detail about the characteristics of the recruited population [4]; involve humans; and [5] be peer-reviewed and published in English between 2008 and 2018.

Papers were excluded if they [1] solely recruited participants with chronic conditions who routinely receive specific medical preconception care (e.g. people with human immunodeficiency virus, epilepsy, type 1 diabetes, cystic fibrosis, severe mental illness, or polycystic ovary syndrome) [2]; solely recruited individuals who were receiving assisted reproductive therapy (ART) as specific preconception care is routinely provided [3]; were retrospective; or [4] were letters to the editors, commentaries, or protocols.

#### Screening process

Duplicates were removed and titles and abstracts, then full texts, were screened by two authors (BH and SC). Double screening was performed on 10% of the papers to establish reliability [17].

#### Data extraction and analysis

Data were extracted by two authors (BH and SC) using a piloted data extraction form which included study aim, explicit and implicit definitions of preconception, participant inclusion and exclusion criteria, participant characteristics, and recruitment methods. Where available, protocol papers or other publications relating to the same study were checked for additional recruitment information. The findings summarised in the data extraction tables were synthesised narratively to create a profile of the characteristics of preconception populations and recruitment methods employed in the included studies.

### Stage 2: concept analysis

The second aim of this study, to generate a working definition of the preconception population and associated attributes, was achieved via concept analysis. A concept analysis provides guidance in understanding a concept of interest that is vaguely defined or poorly understood [18]. It involves the dissection of a concept into simpler elements to promote clarity and understanding and can help elucidate its meaning or definition. Our concept analysis of the term “preconception population” was guided by the eight-step method described by Walker and Avant, which included selecting a concept, determining the aims of the analysis, identifying possible uses of the concept, determining the defining attributes, identifying model cases, identifying additional cases, identifying antecedents and consequences, and defining empirical referents [16]. Descriptions of these steps are outlined in Table 1.

**Table 1:** Walker and Avant’s eight steps of concept analysis with applied methodology

Steps of concept analyses as defined by Walker and Avant (2011)	Definition of steps of concept analyses as defined by Walker and Avant (2011)	Methodology applied
1. Select a concept.	Choose a concept to explore.	Concept chosen by authors discussing variants of terminology describing preconception populations.
2. Determine the aims of the analysis.	Determine the aims or purposes of the analysis.	Aims of the analysis were decided upon by the authors considering the available literature and applying their research experience.
3. Identify all of the possible uses of the concept.	Identify as many uses of the concept as you can find.	After conducting the literature searches and applying inclusion/exclusion criteria, authors extracted all terms and definitions used to explain the targeted or included population. Authors combined and considered all terms and definitions to generate an agreed, general use (or uses) of the concept ‘preconception population’.
4. Determine the defining attributes.	Identifying they key attributes that are most frequently associated with the concept.	Extraction of all described characteristics of included or target populations from inclusion criteria and results of papers. Authors combined and considered all defining attributes and generated the key defining attributes of the preconception population.
5. Identify model cases.	A model case which represents a pure example of the concept or a paradigmatic example which demonstrates all the concept’s defining attributes.	Extraction of the key defining attributes of included or target population. Authors combined and considered all defining attributes and generated hypothetical case studies to represent model cases of a preconception population.
6. Identify borderline, related and contrary cases.	Identifying, examining and defining cases which slightly differ or are contrary to the concept. *Borderline cases:* examples that contain most of the defining attributes of the concept but not all of them. *Related cases:* instances of concepts related to the concept under investigation but do not include all the defining attributes. *Contrary cases:* clear examples of ‘not the concept’.	Extraction of exclusion criteria and consideration of any potentially ineligible characteristics. Authors combined all additional cases and generated hypothetical case studies of borderline, related and contrary cases.
7. Identify antecedents and consequences.	Recognising the circumstances which must/can occur before (antecedent) or after (consequences) the occurrence of the concept. A defining attribute cannot be an antecedent or consequence.	Using the extracted participant characteristics and inclusion/exclusion criteria, the authors considered all potential antecedents or consequences relevant to a preconception population. Authors combined these to generate the key antecedents and consequences of the preconception population.
8. Define empirical referents.	Phenomena that occur or are means by which key attributes of the concept can be measured. In more abstract concepts an example might be “kissing” being an empirical referent for “affection”.	Discussion of the key referent points that allow definition and use of the concept of preconception populations.

#### Literature search

In addition to the included papers from Stage 1, an additional search of relevant publications was performed to provide rich and comprehensive data and ensure that seminal and other key papers on the topic were considered. The additional search included [1] searching relevant documents and media outlets including national guideline bodies in key developed countries (e.g. UK, US, Canada, Australia) such as the UK National Institute for Health Excellence (NICE) guidelines and ‘The Conversation’ (an independent source of news and views, sourced from the academic and research community and delivered direct to the public), and [2] conducting a general search of preconception and relevant terms (as described above in Stage 1) on CINAHL and Google Scholar, without restriction to the other inclusion and exclusion criteria. Relevant papers known to the authors were also included at this stage (including papers pertaining to the US CDC and Select Panel on Preconception Care [7]). For inclusion, documents were required to be identified in Stage 1 or [1] published in English, [2] mention preconception (or similar term) in the title or abstract/summary, and [3] from non-commercial sources.

All potentially eligible papers and documents were read in full by two authors (BH and SC) and the 10 most relevant and seminal papers/documents were selected for inclusion. This decision was made by two authors (BH and SC) independently choosing their top 10 papers/documents then discussing their choices and agreeing on the final papers and documents.

Two authors (BH and SC) independently extracted data relating to the eight steps from all papers identified as eligible in Stages 1 and 2. Subsequently, the authors collaboratively generated a summary of the concept analysis findings, and using the concept analysis methodology, developed a reflective, literature-informed definition of a preconception population.

## Results

### Stage 1: identifying common characteristics of preconception populations

#### Search and screening process

**Fig. 1:**
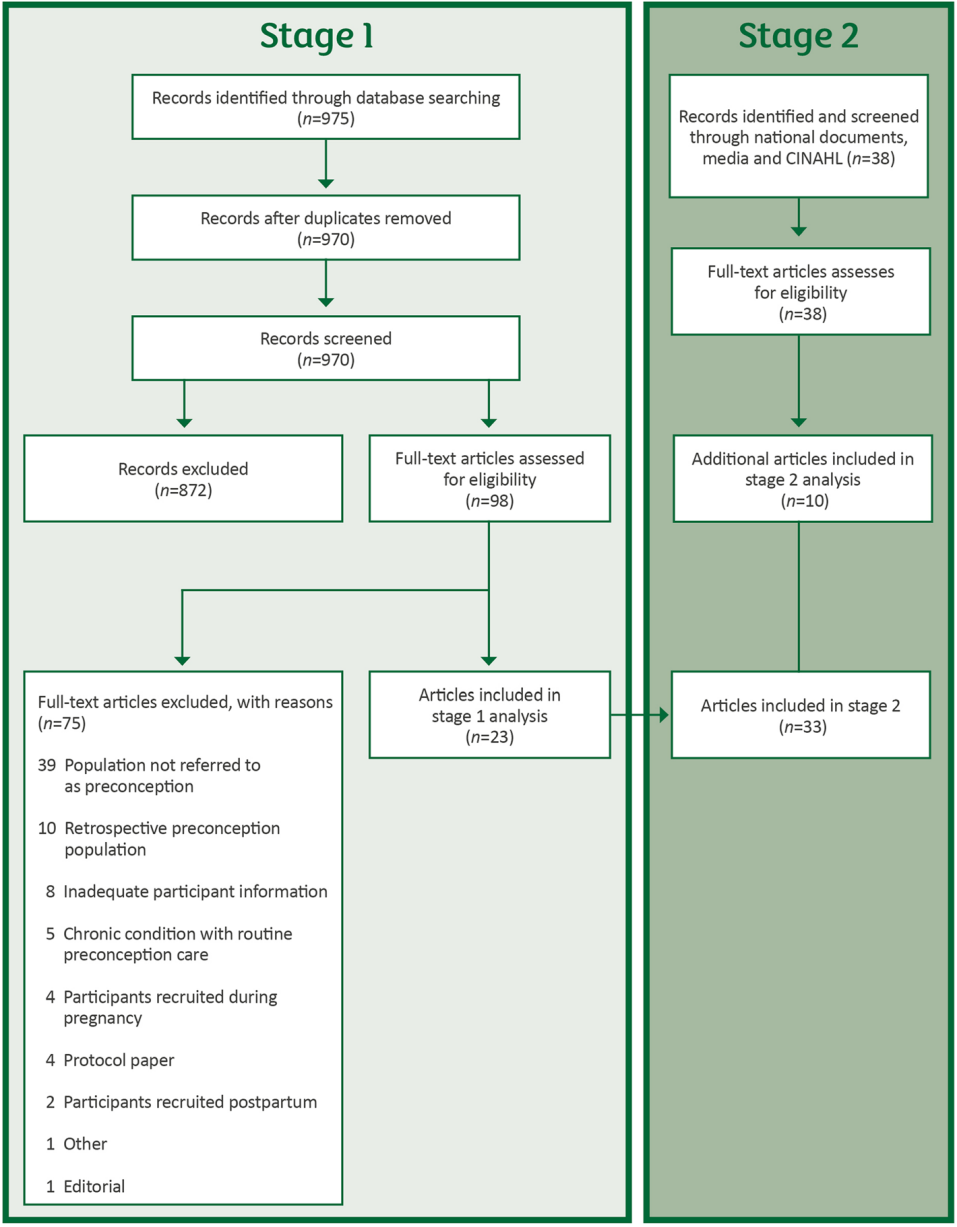
Flow of studies at Stage 1 and Stage 2

The search was conducted in April 2018. Flow of included studies is presented in Fig. 1. Titles and abstracts of 970 papers were screened (with good reliability achieved on a 10% double screened subsample; kappa = 0·8) [17]. Ninety-eight papers were eligible for full text screening (kappa = 0·6 for 10% subsample). Twenty-three papers representing 19 studies were eligible for inclusion. Details pertaining to study characteristics and recruitment methods are presented in Supplementary File 1 and summarised below.

#### Definitions of preconception

Only one study explicitly defined the preconception population, defined as “women with desire to conceive” [19]. Implicit definitions were similar in theme, primarily centring on intentions for pregnancy or current pregnancy plans. One study focused on women and couples of childbearing age [20], whilst another focused on couples with fertility problems [21].

#### Inclusion and exclusion criteria

Regarding inclusion criteria of participants, most studies specified recruitment of women only (*n* = 13), with one solely recruiting men [22]. Age criteria varied, albeit most (*n* = 12) studies recruited participants aged 18 to 45 years. Twelve studies specified that participants must be planning a pregnancy, including specific time scales such as within 1 year, sometime in the future or currently trying to become pregnant. Relationship status was an explicit criterion in three studies, with two allowing only married participants (conducted in Iraq and Vietnam) [23, 24] and one specifying that participants must be in a stable relationship [25]. Many studies also stipulated health or reproductive history criteria such as absence of fertility or reproductive issues (*n* = 4) [25–28], no health risk factors (*n* = 2) [29, 30], receipt of preconception care consultations (*n* = 2) [19, 31], being on a waiting list for in vitro fertilisation (*n* = 1) [21], and previous miscarriage (*n* = 2) [32, 33]. Exclusion criteria were less specified, however these included ongoing pregnancy or breastfeeding (*n* = 5) [20, 24, 26, 31, 34], history of infertility or adverse pregnancy events (*n* = 4) [20, 26, 31, 32], multiple pregnancy (*n* = 1) [29], and contraceptive use (*n* = 1) [26].

#### Participant characteristics

A total of 12,427 participants (131 males and 12,296 females) were included; mean age ranged from 25·4 to 36·7 years (range 14–42). Reporting of other demographic variables was inconsistent, hence data are reported only from the studies that provided information: 669 participants were described as having no children, ranging from 9 to 87% in individual studies and 4541 had one or more children; 5700 were married or living with partner, ranging from 64 to 98% in studies; 704 reported white ethnicity (37–95%); 2657 were employed (35–98%); and 1711 had been educated to university level (7–86%) and 633 to secondary education level (31–66%; see Additional File 1).

#### Recruitment methods

Studies provided varying detail regarding their recruitment methods. Clinic/hospital settings included premarital clinics (*n* = 2) [23, 30], preconception clinics (*n* = 2) [21, 31], obstetric/gynaecological clinics (*n* = 2) [20, 29], general hospital setting (*n* = 1) [32], health centres (*n* = 2) [24, 32], ART clinic (*n* = 1) [20], and a women’s clinic (*n* = 1) [35]. Recruitment from the general population occurred primarily via the Internet/websites (*n* = 5) [22, 25, 26, 34, 36], social media (*n* = 5) [20, 22, 25, 34, 35], posters/flyers in the community (*n* = 3) [20, 25, 26], television, print or radio media (*n* = 2) [25, 26], or word of mouth (*n* = 2) [20, 36].

### Stage 2: concept analysis

#### Steps 1 and 2: select a concept and determine the aims or purposes of analysis

Our concept of interest was the “preconception population” and the purpose of this analysis was to generate a working definition of a preconception population focused on attributes, using concept analysis principles and existing literature.

#### Step 3: uses of the concept

A summary of the included studies and papers as they pertain to Steps 3 to 8 are included in Additional File 2. These relevant documents included the report *Making the Case for Preconception Care* published by Public Health England in 2018 [37]; a chapter on preconception care from the *Family-Centered Maternity and Newborn Care: National Guidelines* published by the Public Health Agency of Canada in 2017 [9]; a consensus statement from the Clinical Workgroup of the National Preconception Health and Health Care Initiative (USA) [38]; a 2017 statement on pre-pregnancy counselling developed by the Royal Australian and New Zealand College of Obstetricians and Gynaecologists [39]; and various reviews published in academic literature [11, 15, 40].

There was a notable absence of the concept of the “preconception population” in the literature, however the term “preconception” was broadly used in the context of women’s health before or around pregnancy or conception. For example, some documents focused on preconception *care* and related needs [41, 42], while others were focused on preconception *health* as a concept [11, 22]. Studies tended to define preconception as a time period rather than a population, for example referring to women before conception [33] or before a pregnancy [24, 28], or using “time to pregnancy” as a measure of the preconception period [33].

#### Step 4: defining attributes

**Fig. 2:**
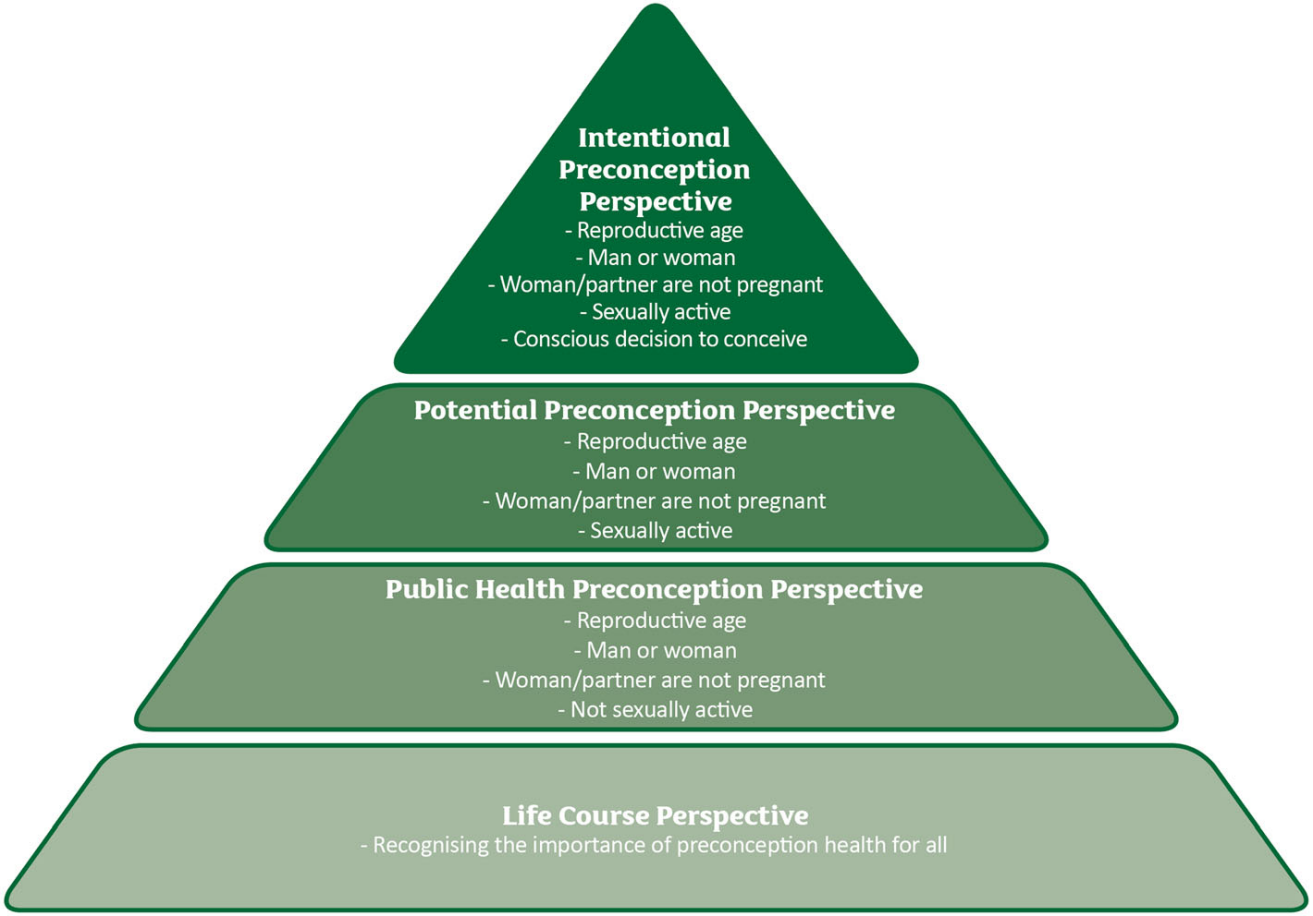
The four perspectives upon which to define preconception populations

Three defining attributes were present for all cases of preconception participants. These were (1) reproductive age; (2) man or woman; (3) woman or partner were not pregnant. These attributes were encompassed across the literature^e.g.,11,40^ and include the majority of the adult population, therefore we broke this down into four definitions where more specific attributes could be applied (see Fig. 2). Firstly, the *public health preconception perspective* includes the three defining attributes described above, but includes only individuals who are not sexually active. The *potential preconception perspective* includes three of the four defining attributes in the public health perspective (reproductive age, man/woman, not pregnant), however, the criteria for not being sexually active is changed to include only individuals who are sexually active, including those who partake in intercourse without using effective contraception or who experience contraceptive failure. This encompasses men or women who may go on to experience unplanned pregnancies. The *intentional preconception perspective* includes the four potential preconception population defining attributes, plus a conscious decision to conceive and/or an element of pregnancy planning. In this way, the intentional perspective focuses on women or men with pregnancy intentions, whether or not specific behavioural changes have been made towards preparing for pregnancy. The *life course perspective* recognises that preconception health can be addressed throughout the life course by targeting populations that do not meet the criteria for the other definitions, for example adolescents or pregnant women. One final approach should be considered that is cross-cutting across the other perspectives; a systems approach to addressing preconception health is essential to support activities across all other perspectives and includes adequate policies and guidelines regarding lifestyle and health; these were reflected in many of the included documents. All of these perspectives apply equally to men and women, to same sex couples and to solo mothers by choice.

#### Step 5: model cases

Examples of model cases for the identified perspectives were created through discussion and reference to existing literature and are presented below. Each one contains the defining attributes. Consider the following example model cases:

Public health preconception perspective:

*“Toni is 26 years old, is not pregnant, and is not currently sexually active.”*

Potential preconception perspective:

*“Toni is 26 and not pregnant. She is not currently planning a pregnancy, but she is sexually active. However, she regularly forgets to take her oral contraceptive pill and sometimes her partner does not wear a condom when they have sex.”*

*“Jaime is 19. He is regularly sexually active with his new girlfriend who is not pregnant. They are careful and use a condom every time.”*

Intentional preconception perspective:

*“Toni is 26 and not pregnant. After discussion with her partner, she has decided that they would like to start trying to have a baby this year. She has stopped taking her oral contraceptive pill and she and her partner deliberately do not use condoms when they have sex.”*

*“Jaime is 19 years old. He is regularly sexually active with his new girlfriend who is not pregnant. They have decided they would like to get pregnant as soon as possible.”*

#### Step 6: borderline, related, and contrary cases

There were some instances where studies recruited or referred to populations that did not meet the defining attributes of a preconception population. A *borderline* case contains most, but not all, of the defining attributes of the concept. The following constructed example illustrates a borderline case from a potential perspective. Here, Sophia has not indicated a specific future pregnancy intention or any pregnancy planning, but might be considered preconception.

*“Sophia is 20, not currently pregnant, is not currently sexually active, but is due to marry soon.”*

A *related* case occurs when a concept is related to the concept of interest but does not contain all defining attributes. The following constructed example may more closely align with the concepts of preconception care [43] or reproductive life planning [12], but does not contain all the defining attributes for a preconception population from an intentional perspective.

*“Jo is 27. She is not currently pregnant but recently gave birth to her first child. Jo and her partner have resumed intercourse after Jo’s recovery from labour and delivery. They think they would like to try for a sibling for their daughter but would like to wait a bit longer and are using condoms when they have sex. Nevertheless, Jo is careful about her reproductive health and has made an appointment at a preconception care clinic to ensure she is healthy and to make any necessary behavioural changes or preparations for future pregnancies.”*

A *contrary* case is a case that does not meet any of the defining attributes (“not the concept”). The following constructed example is a contrary case for both the potential and intentional perspective because Sarah is not currently partaking in intercourse, experiences a permanent form of contraception and is not planning a future pregnancy.

*“Sarah is 32. Recently, after giving birth to her first child, she had medical complications which resulted in a hysterectomy meaning she can no longer become pregnant. She is abstaining from sex with her partner while she recovers.”*

#### Step 7: antecedents and consequences

From the public health perspective, the antecedent to preconception would be childhood or puberty. From a potential perspective, the antecedent is when one becomes sexually active. From an intentional perspective, the antecedent is the decision and action of stopping the use of contraception and/or the act of making a decision that results in a pregnancy intention. Antecedents could be considered flags to identify individuals who may become preconception in the near future.

For the concept of the “preconception population”, there are two consequences. Firstly, a woman (or female partner) becomes pregnant. The second alternative is that the individual exits reproductive age, for example after going through menopause. In the literature, only the consequence of pregnancy was considered in studies which followed women until they reported a pregnancy [33].

#### Step 8: empirical referents

In the concept of the preconception population, intercourse and the use of contraception may be easily measured by asking a simple question. Pregnancy planning (intentional perspective) is often retrospectively assessed via a single item (e.g., “was your pregnancy planned?” [44]) but can be assessed more comprehensively using the London Measure of Unplanned Pregnancy (LMUP) [45]. The LMUP is a 6-item measure that encompasses contraceptive use, feelings about pregnancy, pregnancy intentions (including discussions with partner) and health actions to prepare for pregnancy. However, the LMUP is designed for use during or after pregnancy, and therefore is retrospective. Whether considering the preconception population from a public health, potential, or intentional perspective, there is a need for a validated scale to assess prospective pregnancy intentions. Key opportunities to assess intentions regarding a future pregnancy include adolescents learning about preconception health and family planning during sexual education classes in school, when women visit a medical practitioner to talk about contraceptive use or for cervical screening, when women marry or attend preconception care clinics or in the postnatal period. A new, validated measure that could be applied in these contexts is the Desire to Avoid Pregnancy scale, a 14-item tool that explores attitudes towards pregnancy across cognitive, affective and practical domains [46].

## Discussion

The overall aim of this paper was to explore the concept and attributes of the preconception population and generate a working definition based on the characteristics and recruitment methods identified in the literature coupled with a concept analysis guided by Walker and Avant’s [16] framework. Analysis of the included studies confirmed that preconception populations are poorly and inconsistently defined and described in the literature. Our concept analysis revealed four key perspectives of a preconception population: public health, potential, intentional, and life course (see Fig. 2). Each of these is applicable in certain contexts and can be applied prospectively, enabling researchers, healthcare professionals, and policy makers to proactively address preconception health behaviours and care in these populations.

The public health preconception perspective provides a broad and encompassing definition of a preconception population, which would be most suited to population wide research. Despite variation in participant demographic characteristics and recruitment methods, the majority of included studies defined their preconception populations as reproductive-aged. Interestingly, there was little consideration of males. This is noteworthy given the biological and psychological influence men can have on the health of a pregnancy and child [47, 48]. Furthermore, while the literature was focused on women, and to a lesser extent men, this definition can include other gender identities that are capable of supporting reproduction through provision or donation of egg or sperm. The public health preconception definition would facilitate large, population level research and subsequent, long-term health promotion interventions that captures individuals who do not consider preconception an important life phase to them [15]. We decided to limit our public health perspective to the four defining attributes of reproductive age, man or woman, not being pregnant, and not sexually active. This differs from Stephenson et al. [15] who posit that the public health perspective captures other sensitive phases of the life course such as adolescence. Our life course perspective captures these populations.

The potential preconception perspective includes individuals who are sexually active, including those that have intercourse without using effective or appropriate contraception. This perspective recognises the increased likelihood of pregnancy due to the behaviour of intercourse, however does not include any explicit cognitive considerations of pregnancy planning. This group necessarily incorporates women or couples that are not planning a pregnancy. A key criticism and barrier faced by researchers in the preconception field is how to recruit both individuals who are planning and not planning a pregnancy. In Stage 1, studies were very much focused on those who were planning pregnancy and this was reflected in the studies’ recruitment strategies. It is possible that the challenge of recruiting research participants who are not planning a pregnancy has been a deterrent or barrier to research in this population. With up to 50% of pregnancies unplanned [49, 50], a large group of individuals who are ‘at risk’ of pregnancy are not captured in this research, limiting our understanding of these individuals and potentially excluding them from key health support or interventions. This possible exclusion is problematic as unplanned pregnancies are associated with adverse maternal and infant outcomes [51–53]. While public health initiatives can attempt to reduce the incidence of unplanned pregnancies (and are indeed essential), adopting and considering this potential preconception perspective may allow preconception research and health promotion to reach this at-risk population.

The intentional preconception perspective is identifiable by the addition of the conscious decision to conceive and/or pregnancy planning activities. This is perhaps the most recognisable definition of a preconception population, as was evident in the types of studies that were identified in the search. In many cases, individuals planning pregnancy seek out preconception care of their own volition and may be more likely to engage in healthy behaviours or cessation of unhealthy behaviours (e.g., smoking [54]). Pregnancy planners may be easier to target for research and intervention.

The life course preconception perspective is also important to consider. Specifically, the other three perspectives do not fully capture the breadth of preconception populations and the strategies needed to reach individuals on the periphery. The primary studies included in this concept analysis did not address this broader perspective, which incorporates a systems approach to addressing preconception health, as well as targeting individual behaviour change at life phases that are not captured elsewhere. Systems approaches are widely recognised as essential to achieving reductions in adverse public health problems such as smoking and obesity [55, 56] and have also been applied to obesity prevention across the preconception, pregnancy, postpartum, and early life phases [57]. Stephenson et al. highlight that health behaviours may become established before reproductive age and thus one of the challenges of improving preconception health is to reach women and men across the life course [15]. For example, reaching adolescents or those who are newly sexually active will ensure that individuals are targeted as they transition into a preconception population. We also acknowledge that the systems approach can and should be applied across all of the preconception definitions identified.

Despite the potential use of the preconception definition and essential attributes outlined herein, it is essential to consider the borderline and related cases that were identified in this analysis. These cases represent the ‘at risk’ groups or ‘near misses’ who may be under researched or do not receive preconception care. For example, in some cultures and countries, being recently married, without any behavioural or pregnancy intention is considered a trigger to receive preconception counselling and/or support. Similarly, in the case of Jo, described above, the preconception population criteria for the intentional perspective are not met, but her efforts to prepare herself for pregnancy clearly identify her as a preconception woman. While the public health definition of a preconception population may capture these women, strategies for this group may not be appropriately tailored to the varying needs within the group. Thus, it is important to think beyond the prescribed definitions of preconception populations when necessary, to ensure that key populations are not excluded.

### Limitations

The findings of this review may be limited by the inclusion of only one database for the search. The CINAHL database was selected based on its diversity of literature across a range of allied health professions and disciplines such as nursing, medicine, nutrition and general health, and because the key word search encompassed broad ideas of preconception populations. This approach was considered appropriate given concept analyses do not require extensive literature searches. Furthermore, in Stage 2, the specific exclusion criteria from Stage 1 (used to achieve specificity within the search) were lifted to capture a broader range of relevant literature. The findings may also be limited by the subjective nature of the concept analysis, which is qualitative and could be affected by inherent and unrecognised bias in the data collection and analysis process. However, the use of the structured framework by Walker and Avant and the systematic search process may have gone some way to mitigating this limitation. Furthermore, the concept analysis was conducted collaboratively among the authors with extensive discussion to ensure varying points of view were considered. This review may also be limited by the state of the literature. Specifically, the search highlighted that there are relatively few studies that actively and prospectively recruit preconception women, possibly because the population has been so hard to define.

An important limitation to consider is that this concept analysis is the first attempt to ascribe attributes to preconception populations. Consequently, these definitions require refinement and attention to additional perspectives beyond what are available in the published literature. This includes the views of experts in the field, including researchers, practitioners, policy makers, and consumers/public. The next planned step is to conduct a consensus development exercise to make sense of these definitions in the real world.

## Conclusion

We propose definitions of preconception populations across life course, public health, potential, and intentional preconception perspectives. They consider the attributes of men and women who may be classified as being preconception, e.g. planning pregnancy, as well as those who may not identify as preconception, e.g. those using contraception. Adopting these definitions will allow researchers to accurately identify and recruit their target preconception populations and to develop interventions that are appropriately broad or tailored depending on population needs. In addition to using these proposed attribute-based definitions to recruit appropriate preconception research populations, they can be considered alongside the definitions proposed by Stephenson et al. for broader public health and health promotion purposes [15]. Governing bodies should ensure that appropriate policies, guidelines and directives consider all preconception perspectives to ensure that key or vulnerable populations are not missed. Importantly, reaching preconception populations may require approaches that overlap across these perspectives. The definitions of preconception populations described will make it easier to understand, reach and improve the wellbeing of preconception individuals, which is essential to promoting general health, facilitating healthy pregnancies, and ensuring the health and wellbeing of future generations.

## Supplementary information

**Additional file 1.** Summary of included studies.

**Additional file 2.** Summary Table for Steps 3 to 8 from Walker and Avant’s (2011) Concept Analysis Framework.

## Data Availability

All data generated or analysed during this study are included in this published article and its supplementary information files.

## Abbreviations

ART: Assisted reproductive technology
CDC: Centers for Disease Control and Prevention
CINAHL: Cumulative index to nursing and allied health literature
LMUP: London Measure of Unplanned Pregnancy
NICE: National Institute for Health and Care Excellence
UK: United Kingdom
US: United States

## References

[1] Pantasri T, Norman RJ (2014). The effects of being overweight and obese on female reproduction: a review. Gynecol Endocrinol.

[2] Rasmussen KM, Yaktine AL. Weight Gain During Pregnancy: Reexamining the Guidelines. Washington, DC: Institute of Medicine, National Research Council; 2013. 20669500

[3] Rong K, Yu K, Han X (2015). Pre-pregnancy BMI, gestational weight gain and postpartum weight retention: a meta-analysis of observational studies. Public Health Nutr.

[4] Hanson M, Bhutta ZA, Dain K, Fuchtner C, Hod M (2018). Intergenerational burden and risks of NCDs: need to promote maternal and child health. Lancet.

[5] Reichetzeder C, Dwi Putra SE, Li J, Hocher B (2016). Developmental origins of disease - crisis precipitates change. Cell Physiol Biochem.

[6] Lindsay AC, Greaney ML, Wallington SF, Mesa T, Salas CF (2017). A review of early influences on physical activity and sedentary behaviors of preschool-age children in high-income countries. J Spec Pediatr Nursing.

[7] Johnson K, Posner SF, Biermann J, et al. Recommendations to improve preconception health and health care --- United States: Centers for Disease Control and Prevention, 2006.

[8] National Institute for Health and Care Excellence. Weight Management Before, During, and After Pregnancy (PH27). National Institute for Health and Care Excellence; 2010.

[9] Public Health Agency of Canada. Family-Centred Maternity and Newborn Care: National Guidelines. Chapter 2: Preconception Care, 2017.

[10] World Health Organization. Meeting to Develop a Global Consensus on Preconception Care to Reduce Maternal and Childhood Mortality and Morbidity. Geneva: World Health Organization; 2013.

[11] Toivonen KI, Oinonen KA, Duchene KM (2017). Preconception health behaviours: a scoping review. Prev Med.

[12] Liu F, Parmerter J, Straughn M (2016). Reproductive life planning: a concept analysis. Nurs Forum.

[13] Edmonds SW, Ayres L (2017). Evolutionary concept analysis of reproductive life planning. J Obstet Gynecol Neonatal Nurs.

[14] Banerjee A, Chaudhury S (2010). Statistics without tears: populations and samples. Ind Psychiatry J.

[15] Stephenson J, Heslehurst N, Hall J (2018). Before the beginning: nutrition and lifestyle in the preconception period and its importance for future health. Lancet.

[16] Walker LO, Avant KC (2011). Strategies for theory construction in nursing.

[17] Terwee CB, Bot SDM, de Boer MR (2007). Quality criteria were proposed for measurement properties of health status questionnaires. J Clin Epidemiol.

[18] Foley AS, Davis AH (2017). A guide to concept analysis. Clin Nurse Spec.

[19] M’hamdi HI, Sijpkens MK, de Beaufort I, Rosman AN, Steegers EAP (2018). Perceptions of pregnancy preparation in women with a low to intermediate educational attainment: a qualitative study. Midwifery.

[20] Bortolus R, Oprandi NC, Morassutti FR (2017). Why women do not ask for information on preconception health? A qualitative study. BMC Pregnancy Childbirth.

[21] Ockhuijsen HDL, Gamel CJ, van den Hoogen A, Macklon NS (2012). Integrating preconceptional care into an IVF programme. J Adv Nurs.

[22] Agricola E, Gesualdo F, Carloni E (2016). Investigating paternal preconception risk factors for adverse pregnancy outcomes in a population of internet users. Reprod Health.

[23] Abbas WAK, Azar NG, Haddad LG, Umlauf MG (2008). Preconception health status of Iraqi women after trade embargo. Public Health Nurs.

[24] Nguyen PH, Lowe AE, Martorell R (2012). Rationale, design, methodology and sample characteristics for the Vietnam pre-conceptual micronutrient supplementation trial (PRECONCEPT): a randomized controlled study. BMC Public Health.

[25] Wise LA, Wesselink AK, Tucker KL (2018). Dietary fat intake and fecundability in 2 preconception cohort studies. Am J Epidemiol.

[26] Chason RJ, McLain AC, Sundaram R (2012). Preconception stress and the secondary sex ratio: a prospective cohort study. FertilSteril.

[27] Lum KJ, Sundaram R, Buck Louis GM (2011). Women's lifestyle behaviors while trying to become pregnant: Evidence supporting preconception Guidance. Am J Obstet Gynecol.

[28] Weisman CS, Hillemeier MM, Chase GA (2008). Women's perceived control of their birth outcomes in the Central Pennsylvania Women's health study: implications for the use of preconception care. Womens Health Issues.

[29] Aranda N, Ribot B, Garcia E, Viteri FE, Arija V (2011). Pre-pregnancy iron reserves, iron supplementation during pregnancy, and birth weight. Early Hum Dev.

[30] Bastani F, Hashemi S, Bastani N, Haghani H (2010). Impact of preconception health education on health locus of control and self-efficacy in women. East Mediterr Health J.

[31] Sardasht FG, Shourab NJ, Jafarnejad F, Esmaily H (2017). The frequency of risk factors associated with pregnancy among women seeking planned pregnancy. J Midwifery Reprod Health.

[32] Ahrens KA, Silver RM, Mumford SL (2016). Complications and safety of preconception low-dose aspirin among women with prior pregnancy losses. Obstet Gynecol.

[33] Vousden NJ, Carter J, Seed PT, Shennan AH (2017). What is the impact of preconception abdominal cerclage on fertility: evidence from a randomized controlled trial. Acta Obstet Gynecol Scand.

[34] Agricola E, Pandolfi E, Gonfiantini MV (2014). A cohort study of a tailored web intervention for preconception care. BMC Med Inform Decis Mak.

[35] Goossens J, Delbaere I, Dhaenens C (2016). Preconception-related needs of reproductive-aged women. Midwifery.

[36] van der Zee B, de Beaufort ID, Steegers EAP, Denktas S (2013). Perceptions of preconception counselling among women planning a pregnancy: a qualitative study. Fam Pract.

[37] Public Health England. Making the Case for Preconception Care: Planning and Preparation for Pregnancy to Improve Maternal and Child Health Outcomes. London: Public Health England Publications; 2018.

[38] Frayne DJ, Verbiest S, Chelmow D (2016). Health care system measures to advance preconception wellness: consensus recommendations of the clinical workgroup of the National Preconception Health and health care initiative. Obstet Gynecol.

[39] Royal Australian and New Zealand College of Obstetricians and Gynaecologists. Pre-pregnancy counselling, 2017.

[40] Thompson EL, Vázquez-Otero C, Vamos CA, Marhefka SL, Kline NS, Daley EM (2017). Rethinking preconception care: a critical, women’s health perspective. Matern Child Health J.

[41] Hall JA, Mann S, Lewis G, Stephenson J, Morroni C (2016). Conceptual framework for integrating ‘pregnancy planning and prevention’ (P3). J Fam Plann Reprod Health Care.

[42] Hemsing N, Greaves L, Poole N (2017). Preconception health care interventions: A scoping review. Sex Reprod Healthc.

[43] Atrash H, Jack BW, Johnson K (2008). Preconception care: A 2008 update. Curr Opin Obstet Gynecol.

[44] Cheng TS, Loy SL, Cheung YB (2016). Demographic characteristics, health behaviors before and during pregnancy, and pregnancy and birth outcomes in mothers with different pregnancy planning status. Prev Sci.

[45] Barrett G, Smith SC, Wellings K (2004). Conceptualisation, development, and evaluation of a measure of unplanned pregnancy. J Epidemiol Community Health.

[46] Rocca CH, Ralph LJ, Wilson M, Gould H, Foster DG (2019). Psychometric evaluation of an instrument to measure prospective pregnancy preferences: the desire to avoid pregnancy scale. Med Care.

[47] Fleming TP, Watkins AJ, Velazquez MA (2018). Origins of lifetime health around the time of conception: causes and consequences. Lancet.

[48] Cheng ER, Rifas-Shiman SL, Perkins ME (2016). The influence of antenatal partner support on pregnancy outcomes. J Women's Health (Larchmt).

[49] Finer LB, Zolna MR (2016). Declines in unintended pregnancy in the United States, 2008–2011. N Engl J Med.

[50] Bearak J, Popinchalk A, Alkema L, Sedgh G (2018). Global, regional, and subregional trends in unintended pregnancy and its outcomes from 1990 to 2014: estimates from a Bayesian hierarchical model. Lancet Glob Health.

[51] Shah PS, Balkhair T, Ohlsson A, Beyene J, Scott F, Frick C (2011). Intention to become pregnant and low birth weight and preterm birth: a systematic review. Mat Child Health J.

[52] Hall JA, Benton L, Copas A, Stephenson J (2017). Pregnancy intention and pregnancy outcome: systematic review and meta-analysis. Mat Child Health J.

[53] Abajobir AA, Maravilla JC, Alati R, Najman JM (2016). A systematic review and meta-analysis of the association between unintended pregnancy and perinatal depression. J Affect Disord.

[54] U.S. Department of Health and Human Services. The Health Consequences of Smoking - 50 Years of Progress: A Report of the Surgeon General. U.S. Department of Health and Human Services, National Center for Chronic Disease Prevention and Health Promotion, Office on Smoking and Health, 2014.

[55] Lee BY, Bartsch SM, Mui Y, Haidari LA, Spiker ML, Gittelsohn J (2017). A systems approach to obesity. Nutr Rev.

[56] Borland R, Young D, Coghill K, Zhang JY (2010). The tobacco use management system: analyzing tobacco control from a systems perspective. Am J Public Health.

[57] Nader PR, Huang TT-K, Gahagan S, Kumanyika S, Hammond RA, Christoffel KK. Next steps in obesity prevention: altering early life systems to support healthy parents, infants, and toddlers. Child Obes 2012;8(3):195–203. 10.1089/chi.2012.0004 22799545

