# Supplementary material for: Defining preconception: exploring the concept of a preconception population
Source: BMC Pregnancy Childbirth. 2020 May 7;20:280. doi: 10.1186/s12884-020-02973-1 (PMC7206804; doi:10.1186/s12884-020-02973-1)
Supplement: Supplementary file 1 — Additional file 1. Summary of included studies. [file 12884_2020_2973_MOESM1_ESM.pdf]

Defining preconception: Exploring the concept of a preconception population

Additional File 1

Briony HILL[1*^], Jennifer HALL[2], Helen SKOUTERIS[1], and Sinéad CURRIE[3*]

[1] Monash Centre for Health Research and Implementation, School of Public Health and Preventive Medicine, Monash University, Level 1, 43-51 Kanooka Grove, Clayton, Victoria, 3168
[2] EGA Institute for Women's Health, University College London, 74 Huntley St, London, WC1E 6AU UK.
[3] Psychology, Faculty of Natural Sciences, University of Stirling, United Kingdom, FK9 4LA.

*Authors contributed equally

^Address for correspondence:
Dr Briony Hill
Monash Centre for Health Research and Implementation
Level 1, 43-51 Kanooka Grove, Clayton, Victoria, 3168
E: briony.hill@monash.edu

Additional File 1. Summary of included studies.

**Study Author (year)**	**Country**	**Study aim/ rationale**	**Preconception definition: Explicitly stated**	**Preconception definition: Implicitly stated**	**Inclusion criteria**	**Exclusion criteria**	**Sample characteristics**	**Recruitment method**
Abbas et al. (2008)	Iraq	Identify risks for infant mortality and poor maternal outcomes among women in the interval just before conception.<br><br>Improving preconception health will improve health of families and communities.	None	Women contemplating pregnancy	Women attending hospital for routine premarital blood tests, the planned marriage is the first for both partners, women were planning to conceive.	None	All women; N=500; 88% unemployed; 21% primary level education or illiterate; 35% intermediate education; 31% secondary education; 12% university education; 56% aged 20-24.9 years; 29% aged 15-19.9 years; 13% aged 25-29.9 years; 3% aged >30 Mean BMI 22.9-24.9 kg/m^2.	Women attending public hospital for routine premarital blood tests; Hospital was chosen randomly from a choice of 7.
Agricola et al. (2014)	Italy	Explore the efficacy of an informative and tailored intervention in a population of Italian women of childbearing age planning a pregnancy.<br><br>Reduce adverse pregnancy outcomes	None	Women of childbearing age who are planning a pregnancy within a year.	Female gender, aged 18–45 years, reside in Italy, Italian language spoken, plan to fall pregnant within the following year, active email address and internet access, online informed consent.	Ongoing pregnancy.	508 (out of 896 were eligible). Mean age (SD) years: 32.4 (4.8); University degree: N=306, 60.2%; Employed: N=426, 83.9%; Planning first pregnancy: N=377, 75%; BMI <18.5kg/m^2 N=34, 6.7%; BMI >25: N=107, 27.3%.	Women recruited on a web platform. Convenience sampling of women consecutively visiting the online platform and requesting to be enrolled. Study promoted on Facebook and articles explaining the project.
Agricola et al. (2016)	Italy	Measure the prevalence of paternal preconception risk factors for adverse pregnancy outcomes in a population of Italian Internet users.<br><br>Paternal preconception	None	Prospective fathers: a man of a couple planning a pregnancy in the following year.	Male, 18 years of age, reside in Italy, partner with an ongoing pregnancy or pregnancy planned in the following year.	None	Prospective fathers: N=131; mean age (SD) years: 35.06 (7.33); University degree: N=53, 40.46%; Employed: N=125, 96.9%.	Data collected on survey monkey; advertised on well-known Italian websites dedicated to preconception, pregnancy, childhood and family care. Project web pages prompted through web

		health and lifestyles correlated to the occurrence of malformations and birth defects in the offspring as well as maternal health status during pregnancy.						pages, social network accounts and newsletters of the host websites.
Ahrens et al. (2016)<br><br>Schisterman et al. (2014)<br><br>Silver et al. (2015)<br><br>Sjaarda et al. (2017)	USA	To determine whether preconception-initiated LDA improves live-birth rates in women with 1–2 prior losses in the Effects of Aspirin in Gestation and Reproduction (EAGeR) trial.<br><br>Low-dose preconception aspirin has been proposed to improve pregnancy outcomes in couples experiencing recurrent pregnancy loss.	None	Women trying to conceive by natural conception.	Women 18-40 years with a history of 1-2 pregnancy losses and who were trying to conceive by natural conception. Presence of intact tubes, ovaries and uterus, regular menstrual periods between 31-42 days in length, not using contraception by the baseline visit.	Women with contra-indications to aspirin, clinical indication for aspirin therapy, major medical disorder, history of infertility of sub-fertility, current or planned ART, known current or recent alcohol or drug abuse.	N=1228;<br>Mean age (SD) years: 28.7(4.8);<br>Mean BMI (SD): 26.3 (6.6) kg/m^2;<br>95% white;<br>98% married or living with a partner;<br>86% educated at more than high school education;<br>76% employed.	Women were recruited through clinical and community- based recruitment via: the University of Utah Health Sciences Center, McKay Dee Hospital, LDS Hospital, and Utah Valley Regional Medical Centre, University at Buffalo Free Standing Women's Health Center, Moses Taylor Hospital at Scranton and University of Colorado.
Aranda et al. (2011)	Spain	To analyse the relationships between iron supplementation administered early or late in gestation, the haematological and biochemical parameters of the mother during pregnancy and the weight of the newborn.<br><br>Early iron supplementation has risks and so these must be evaluated.	None	Women intending to become pregnant soon.	Healthy women volunteers intending to become pregnant soon, aged between 18 and 35 years.	Woman suffering from any chronic illness that would alter nutritional or inflammation status, needing specific dietary/ nutritional treatment, and multiple pregnancy.	N=82; Iron stores (IS) present at preconception n=52; Low iron (LI) stores at preconception N=30;<br>Mean age years (SD), IS: 29.6 (2.8), LI: 29.4 (2.6);<br>Education, secondary IS: 66%, LI: 62.1%; university IS: 16%, LI: 6.9% (defined as middle class by the authors).	Residents of Reus (Spain) attending the Unit of Obstetrics and Gynaecology of St Joan Hospital, Reus.
Bastani et al. (2010)	Islamic Republic of Iran	To evaluate the impact of a health education intervention regarding exercise/PA among Iranian women attending premarital counselling (RCT).<br><br>Preconception lifestyle intervention may help optimise the health of mother-to-be.	None	Intending to conceive in their first year of marriage (currently attending premarital counselling).	Women of childbearing age (18-35 years), healthy with no identifiable health risk factors (self-reported), intending to conceive in their first year of marriage, literate in Farsi.	Medical or health problems during the recruitment period.	N=240<br>Mean age (SD) years: 25.7 (4.8);<br>Education: Diploma/below: 36.7%;<br>University: 63.3%;<br>Employed: 35.2%;<br>Economic status, good: 31.8%;<br>bas/moderate: 68.2%.	A prospective sample of Iranian women who were attending a premarital counselling clinic and met the inclusion criteria. All premarital clinics in West Tehran were included; N=2.

Bortolus et al. (2017)	Italy	To identify barriers and develop appropriate communication strategies to successfully promote PC.<br><br>Improve preconception care and health as a means of reducing risks of adverse pregnancy outcomes focus on obesity.	None	Women and couples of childbearing age.	Women aged 22–44 years, nulliparae planning a pregnancy in the following 2 years, multiparae having at least one child less than 4 years old (so they are not too far from their maternity experience),	Women with an ongoing pregnancy, women working in the healthcare field, women with previous adverse pregnancy events.	N=14; women of childbearing age; 8 nulliparae and 6 multiparae;<br>Mean age for nulliparae: 36.7 years (range: 33–43); multiparae: 34.7 years (range: 23–46); had 1 or 2 children with an average age of 19 months;<br>N=10 women had a university degree;<br>N=11 married.	Recruited through contacts with associations (N=3); Primary care Paediatricians (N=3);<br>Posters (N=2); Obstetric-Gynaecologists (OBGYNs) of the Verona University Hospital (N=2); The assisted reproduction center (N=2); Facebook (N=1) and personal contacts (N=1).
Chason et al. (2012)	UK	To evaluate the association between psychosocial stress and the 2° sex ratio.<br><br>Explore biomarkers of preconception stress in relation to the 2° sex ratio as this may influence the ratio of male-to-female conceptions.	None	Planning a pregnancy (in inclusion criteria of cohort study)/ women who were discontinuing contraception for purposes of becoming pregnant.	Planning pregnancy or trying for <3 months, aged 18–40 years, and a menstrual cycle length between 21 and 39 days.	History of infertility, current breastfeeding, recent hormonal contraception without intervening menses, or use of injectable contraception any time in the year before enrollment.	N=338;<br>data split into male vs female baby:<br>Mothers' mean age years (30.4 ± 4.2 vs. 29.2 ± 3.5 years.	(Info taken from design paper).<br>Posters in<br>Primary care centres, nurseries, local newspapers and magazines, radio and websites.
Goossens et al. (2016)	Belgium	To explore women's interest in preconception care and its content.<br><br>Poor behaviours prior to conception influencing child health and outcomes. Poor engagement and availability of preconception care.	None	Couples contemplating pregnancy.	Women aged 18-45 with a desire to have (more) children, fluent in Dutch.	None	N=242;<br>Mean(SD) age: 25.4 (4.3) years;<br>86% had a partner;<br>77% had higher level of education;<br>59% employed;<br>87% had no children and wanted children within 3-5 years.	Convenience sample.<br>Posters in women's clinics
Lum et al. (2011)	USA	To prospectively assess women's use of cigarettes, alcohol and caffeine during the preconception period, particularly in relation to women's intentions to modify behavior given interest in becoming pregnant.	None	Women planning a pregnancy.	Ages 18–34 years and no self-reported history of fecundity impairments or infertility.	None	N=90;<br>66% college educated; 76% employed; 98% married; 70% parous; 100% of<br>women self-defined as of white non-Hispanic ethnicity.	Recruited from a previous study.
M'hamdi et al. (2018)	Netherlands	To explore perceptions of pregnancy preparation of women with a relatively low educational attainment and	Women with desire to conceive.	Women with desire to conceive.	Consent to be contacted for an additional study, having received a PCC	None	N=28;<br>Age 24 to 41 years, median 32 years.	Recruitment through another study: Healthy Pregnancy for All (HP4All) Preconception

		the role they attribute to healthcare professionals.<br><br>Increase PCC engagement and pregnancy outcomes.			consultation in 2014, indication for having a low to middle SES based on a low or intermediate educational attainment.			Care study. Recruitment strategy was an invitational letter for PCC from a general practitioner (GP) and/or from the municipality.
Nguyen et al. (2012)<br><br>Young et al. (2015)	Vietnam	To examine whether prepregnancy weekly IFA or MM supplementation improves birth outcomes as well as maternal and infant iron status compared to the current practice of providing only prenatal IFA supplements in the PRECONCEPT trial.<br><br>Improve child health outcomes.	None	Married and not pregnant and intending to become pregnant in the next year.	Aged 18–35 years, currently married, living in one of the 20 communes selected with the intention to stay in the areas for the 24 months following recruitment, plan to have children in the next year, agree to participate with informed consent.	Currently pregnant, regularly consumed IFA or MM supplements in the past 2 months, severe anemia (Hemoglobin [Hb] < 7 g/L; based on testing at enrollment), history of high risk pregnancy, reported chronic hematological diseases, hereditary defects of red blood cells or hemoglobin.	N=5011;<br>Mean (SD) age: 26.2 (4.6) years;<br>Married: 100%;<br>9% no children;<br>88% 1 child;<br>2.5% over one child.	Village health workers visited homes of women on a list from the local health center who were married and not pregnant.
Ockhuijsen et al. (2012)	Netherlands	To study the outcomes of integrating preconception care into an in-vitro fertilization program on nurses' and patients' attitudes and patients' weight and smoking behaviour.<br><br>Improving preconception health will improve effectiveness of IVF.	None	Couples with fertility problems.	Patients on the waiting list for an IVF of ICSI treatment who had visited the preconception clinic and able to read Dutch and nurses who had been counselling patients for at least 3 months.	None	N=130 women;<br>Aged 25-42 years.	Women attending preconception clinic.
Sardasht et al. (2017)	Iran	To determine the risk factors for pregnancy health among females seeking planned pregnancy.<br><br>Identifying risk factors for pregnancy can help prevent maternal morbidity and mortality.	None	Women who referred to health centers to received preconception care.	Having Iranian nationality, and living in Mashhad, reproductive age, referring to health centers to receive preconception counselling, speaking Persian, have at least a	Being a member of the health team, infertility, pregnancy, mental illness and using psychiatric medication at present or in the past.	N=350;<br>Mean age (SD): 26.9 (5.2) years; range 14-41 years;<br>44% attained secondary school as the highest level of education;<br>90.9% participants were housewives;	Health centers were classified into 5 groups and selected for participation. Eligible women referred to the health centers to receive preconception care (In Mashhad) were selected

					minimum literacy level, having physical and mental health.		77% reported a satisfactory income level.	through a convenience sample method.
Szwajcer et al. (2008)	Netherlands	To examine whether preconception and pregnancy can be an occasion triggering interest, search and need for general nutrition information as well as pregnancy-specific nutrition-related information topics.<br><br>Healthy nutrition promotion directed at preconception and pregnancy as a transitional life stage may be a positive exception to the general feeling that healthy behaviour change is difficult to achieve.	None	Women who had stopped using contraceptives in order to become pregnant.	Women aged between 20-40 years, born and raised in the Netherlands, and (if pregnant) had already consulted a midwife. One subgroup contained women who were trying to become pregnant.	Women seen exclusively by a Gynaecologist.	N=422, trying to conceive N=100.<br><br>Age mean (SD): 31.4(0.42) years;<br>98% working;<br>56% high education level.	Women approached through access panels of market research organisations.
Van der Zee et al. (2013)	Netherlands	To analyse women's attitudes and norms with respect to the preconception period and preconception consultation.<br><br>It is unclear what women this of preconception care in general and how they perceive preconception care to fit in their personal situation.	None	Considering pregnancy.	None	None	N=16;<br>Aged between 22-39 years (Mean: 32.8 years);<br>Education:<br>Low n=3, Medium n=3, High n=10;<br>Ethnicity:<br>Dutch n=12, Moroccan n=2, Surinamese n=2.	Women were recruited through the internet, via a network of ethnic minority women, and by use of snowball recruitment, responding to a general invitation.
Vousden et al. (2017)	UK	To determine whether placement of abdominal cerclage before conception affects rates of fertility.<br><br>There are no RCTs comparing effectiveness pf preconception vs. expectant cerclage or the effect on fertility.	None	Women who registered for the trial before conception.	Women with a previous second-trimester miscarriage or preterm birth before 28 weeks of gestation despite having a low vaginal cerclage in place.	Women unwilling to give consent or <16 years of age.	N=67;<br>Mean age (SD): Intervention group: 31(3.9) years,<br>Control group = 32 (4.2) years;<br>Mean (SD) BMI: intervention group: 32 (5.2) kg/m^2, Control group: 28 (5.0) years;<br>Ethnicity (%), white - Intervention group: 37%, Control group 44%.	None

Weisman et al. (2008)	USA	To examine non-pregnant women's beliefs about whether or not they can influence their future birth outcomes with respect to their babies' health (Central Pennsylvania Women's Health Study; CePAWHS).<br><br>It is currently not known whether women contemplating pregnancy believe that they can increase the likelihood of having a healthy baby through their own actions.	None	Considering becoming pregnant at some time in the future.	Women aged 18-45, residence in 28-county target region, English or Spanish speaking. Analytic sample was based on women with reproductive capacity (no reported hysterectomy, tubal ligation or infertility), and pregnancy intent (sometime in the future).	Participants needing a proxy or translator to respond to the survey.	N=614;<br>Age: 90% 18-34; 10% 35-45 years;<br>Race/ethnicity: 87% white/non-Hispanic;<br>64% married or living with partner;<br>76% employed part-time or full-time.	A random-digit dial telephone survey with over sampling in rural counties and areas estimated to include at least 30% minority populations.<br>The study was publicised in the media and through community organisations, and sample households were prenotified with a letter and $2 incentive.
Wise et al. (2018)	Denmark (“Soon Parents” [SF])<br><br>North America (United States or Canada) (Pregnancy Study Online [PRESTO])	To examine total dietary fat intake and intakes of major subtypes of fatty acids, in relation to fecundability in two cohort studies.<br><br>Fat rich diets may be associated with infertility, however further research is needed.	None	Attempting to become pregnant.	SF: 18-45 years, residents of Denmark, in a stable relationship with a male partner, planning a pregnancy, and not receiving fertility treatment.<br><br>PRESTO: 21-45 years, residents of US or Canada, then the same as SF.	Analyses were limited to women who had been trying to conceive for 6 cycles or fewer.	SF: N=1126;<br>PRESTO: N=1290;<br>Demographic characteristics are presented via dietary fat intake for quartiles 1 and 4 for each study.<br><br>% Energy from total dietary fat PRESTO Q1:<br>Age (SD) 29.9(4.0) years; Q4: Age (SD): 30.6 (3.8) years.<br><br>SF Q1:<br>Age (SD): 28.3 (4.3) years;<br>Q4 age (SD): 28.0 (4.3) years.	SF: Internet-based recruitment with advertisements on Danish health-related websites and blogs.<br><br>PRESTO: Internet-based recruitment (study modelled after SF), including banner advertisements on social networking sites (e.g. Facebook), health-related websites, pregnancy-related websites and parenting blogs;<br>Flyers in local shots and community centers;<br>Advertising in print magazines and newsletters; Postcards to newly married women who were on public mailing lists.
