# Supplementary material for: Defining preconception: exploring the concept of a preconception population
Source: BMC Pregnancy Childbirth. 2020 May 7;20:280. doi: 10.1186/s12884-020-02973-1 (PMC7206804; doi:10.1186/s12884-020-02973-1)
Supplement: Supplementary file 2 — Additional file 2. Summary Table for Steps 3 to 8 from Walker and Avant’s (2011) Concept Analysis Framework. [file 12884_2020_2973_MOESM2_ESM.pdf]

Defining preconception: Exploring the concept of a preconception population

Additional File 2

Briony HILL[1]*^, Jennifer HALL[2], Helen SKOUTERIS[1], and Sinéad CURRIE[3]*

[1] Monash Centre for Health Research and Implementation, School of Public Health and Preventive Medicine, Monash University, Level 1, 43-51 Kanooka Grove, Clayton, Victoria, 3168
[2] EGA Institute for Women's Health, University College London, 74 Huntley St, London, WC1E 6AU UK.
[3] Psychology, Faculty of Natural Sciences, University of Stirling, United Kingdom, FK9 4LA.

*Authors contributed equally

^Address for correspondence:
Dr Briony Hill
Monash Centre for Health Research and Implementation
Level 1, 43-51 Kanooka Grove, Clayton, Victoria, 3168
E: briony.hill@monash.edu

Additional File 2. Summary Table for Steps 3 to 8 from Walker and Avant's (2011) Concept Analysis Framework*

Study/type	*Step 3*	*Step 4*	*Step 5*	*Step 6*	*Step 7*		*Step 8*
	**Relevant uses of the concept**	**Observed defining attributes**	**Observed aspects of "model cases"**	**Observed aspects of borderline, related, contrary, invented and illegitimate cases**	**Identified antecedents**	**Identified consequences**	**Observed empirical referents**
Abbas et al. (2008)<br><br>Peer reviewed	Preconception counselling.	Marriage<br>young woman<br>before conception.	Young woman before pregnancy.		Health of mother and baby and ultimately the impact on the economy.<br><br>In good health before pregnancy.	Less/ no pregnancy complications.<br><br>Healthy baby.<br><br>Able to nurse (breastfeed)	
Agricola et al. (2014)<br><br>Peer reviewed	Preconception care.	Women<br>child bearing age<br>planning a pregnancy within the next year.	Women of<br>child bearing age planning a pregnancy within the next year.		Planning a pregnancy in next year.	Reduce risks of adverse pregnancy outcomes.	
Agricola et al. (2016)<br><br>Peer reviewed	Preconception interventions.<br><br>Paternal preconception health.<br><br>Prospective fathers.	Men in a couple<br>planning a pregnancy in following year.	Male with a female partner who plan to become pregnant in next year.	Partner is currently pregnant.	Planning a pregnancy in next year.	Health behaviours and health before conception<br>reduce incidence of adverse pregnancy outcomes.	
Ahrens et al. (2016)<br><br>Schisterman et al. (2014)<br><br>Silver et al. (2015)<br><br>Sjaarda et al. (2017)<br><br>Peer reviewed	Preconception<br><br>Before pregnancy.<br><br>Prior to conception.	Women<br>reproductive age<br>trying to conceive by natural conception.	Women activity trying to become pregnant.	No active intention/ behaviour to become pregnant.		Reduce risk of preeclampsia and other negative reproductive outcomes.<br><br>Incidence of live birth	
Aranda et al. (2011)<br><br>Peer reviewed	Prior to conception.<br><br>Women intending to become pregnant soon.	Women aged between 18 and 35 years.<br><br>Trying to become pregnant soon.		Infertility<br><br>Want to become pregnant.		Conception<br><br>Effect of lack of prepregnancy iron on the newborn.	
Bastani et al. (2010)<br><br>Peer reviewed	Preconception period is an ideal opportunity	Childbearing age<br>(18-35 years).	Before becoming pregnant for the first time.		Highly motivated (if planning pregnancy).	Consequences of overweight and/or underweight on pregnancy	

	to optimise the health of the mother-to-be.<br><br>Pre-marital counselling.	Intending to conceive in the first year of marriage.				and pregnancy outcomes.	
Bortolus et al. (2017)<br><br>Peer reviewed	Preconception care<br><br>Preconception health.<br><br>Health of women and couples of childbearing age.	Child bearing age<br>women and/or couples before pregnancy.<br><br>Planning a pregnancy within 2 years.		Currently pregnant.		Reduce risks of adverse reproductive outcomes.<br><br>Improve health and behaviours.	
Chason et al. (2012)<br><br>Peer reviewed	Preconception (stress).	Women discounting contraception for the purposes of becoming pregnant.<br>reproductive age.		Infertility.<br><br>Use of contraception.			
Frayne et al. (2016)<br><br>Consensus statement	Preconception wellness - a women's overall health before conception.<br><br>Both actively desiring pregnancy and those who become pregnant unintentionally.	Health status before a pregnancy.<br><br>Women of reproductive age.		Delivery of preconception care.<br><br>Recognition that multiple health care providers, clinics, and public health programs deliver preconception care.<br><br>Women with chronic conditions requiring specific preconception care to achieve optimal control before conception.<br><br>Intended/unintended pregnancies or pregnancy planning	PC Health status may be affected by many different service points within the health care system.<br><br>Previous pregnancy (i.e. interpregnancy/inter-conception)	Preconception wellness achieved and preconception care available.<br><br>Entering pregnancy<br><br>Preconception health/wellness will impact baby's health.	A consensus statement - consensus recommendations of the Clinical Workgroup of the National Preconception Health and Health Care Initiative (ACOG).
Goossens et al. (2015)<br><br>Peer reviewed	Before conception.<br><br>Preconception care.	Women (mainly) and couples before contraception<br>reproductive age<br>desire to have (more) children.	Women of reproductive age with desire for pregnancy.		Healthier pregnancy reduce risk of maternal and childhood mortality and morbidity.	Reduce risk of adverse reproductive outcomes.	Preconception care is a form of primary prevention and can be defined as 'the provision of biomedical, behavioural and social health interventions to women and couples before conception<br>occurs' (World Health Organization,<br>2013).
Hall et al. (2016)<br><br>Letter to the editor	Reproductive needs of women and their partner.	Consideration of pregnancy intention:<br>1) desire for pregnancy,<br>2) unsure of pregnancy intention or<br>3) no desire for pregnancy	Individual level attribute (as compared to service and policy level).	Wishing to prevent pregnancy.		Pregnancy outcomes<br><br>Pregnancy	

	Focus on preconception care needs.		Consideration of pregnancy intention (don't need to want to become pregnant).	Planning to become pregnant.<br><br>Those with pre-existing conditions (e.g., diabetes, obesity HIV) that may affect fertility or pregnancy outcome.<br><br>Preconception care.<br><br>Desires pregnancy, unsure or does not desire pregnancy.		Family planning<br><br>Preconception care<br><br>Planning/ preparing for a pregnancy hence having a child by choice.	
Hemsing et al. (2017)<br><br>Peer reviewed scoping review	Women and Men.<br><br>Focus on preconception care	Women and men<br><br>Reproductive years/ age	Risk assessment, preconception and interconception advice should be given to all women and men, not just those identified as being at risk.	Preconception care<br><br>Planning pregnancy<br><br>At risk of poor maternal or child health outcomes.<br><br>Men/women/couples with chronic disease.<br><br>Life course approach to preconception health.	Preparing for parenting.<br><br>Contraception	Parenting<br><br>Fetal health	
Lum et al. (2011)<br><br>Peer reviewed	Women trying to become pregnant.<br><br>Preconception guidance.	Women attempting to become pregnant.	Women of reproductive age with an intention to become pregnant.		Pregnancy intention.	Healthy lifestyles before and during pregnancy.	
M'hamdi et al. (2018)<br><br>Peer reviewed	Pregnancy preparation.<br><br>Preconception health.<br><br>Periconception period.<br><br>Preconception care.<br><br>Actively preparing for a pregnancy	Actively preparing for a pregnancy.<br><br>Women<br><br>Reproductive age.	No pregnancy planning or preparing.		Desire to conceive.	Reduce poor pregnancy outcomes and risk of non-communicable disease in the long term.	Periconception period, defined as the fourteen weeks before and ten weeks after conception, due to the processes of gametogenesis, organogenesis and placental development. (Steegers-Theunissen et al. 2013).
Nguyen et al. (2012)<br><br>Young et al. (2015)<br><br>Peer reviewed	Preconceptual<br><br>Before pregnancy<br><br>Pre-pregnancy	Reproductive age<br>married (cultural)<br>plan to have children in next 1 year.				Reduce low birth weight and anemia.<br><br>Improve birth outcomes.	

						Improve maternal and infant iron status (linked with intervention).	
Ockhuijsen et al. (2012)<br><br>Peer reviewed	Preconceptional care.	Women and couples awaiting IVF treatment (implicit intention for pregnancy).		Not on IVF waiting list.	Fertility issues	Improved health of couples trying to get pregnant.	
Public Health England (2018)<br><br>Report	Women and men of reproductive age/ during reproductive years.<br><br>Period of time before a woman becomes pregnant.<br><br>Prior to pregnancy.	Reproductive age/ reproductive lifecourse.<br><br>Women and men<br><br>Weeks to months before pregnancy begins.<br><br>Period of time when women are at childbearing age.<br><br>Planning a pregnancy<br><br>Inter-conception	Seeking advice and engaging in positive health behaviours prior to trying to conceive.	Prior to first pregnancy, managing risks, next baby and beyond.	Self-identification of woman and their partners that they want to become pregnant.<br><br>May or may not coincide with cessation of contraception use<br><br>Become a woman of childbearing age.<br><br>Discontinuing contraception<br><br>Miscarriage or abortion.	(In relation to preconception health) maternal, infant and child outcomes.<br><br>Give every child the best start in life.	Planning and fitness for pregnancy (terms used throughout).
Royal Australian and New Zealand College of Obstetrics and Gynaecology (2017)<br><br>Position statement	Pre-pregnancy<br><br>Women's health prior to conception.<br><br>Women planning a pregnancy.	Women<br><br>Prior to pregnancy.<br><br>Planning a pregnancy.	Woman, not pregnant, planning a pregnancy.			Baby's health	
Sardasht et al. (2017)<br><br>Peer reviewed	Preconception care.	Females seeking planned pregnancy.<br><br>Reproductive age.<br><br>History of pregnancy (interconception)		Infertility<br><br>pregnancy		Unwanted pregnancy.<br><br>Abortion	
Skau et al. (2016)<br><br>Peer-reviewed protocol	Lifestyle choices leading to diabetes and non-communicable diseases in PC women 'Women's health prior to pregnancy' i.e. focus on women's preconception health (rather than care)	'Young' women;<br><br>Reproductive age (20-39)<br><br>Women AND their spouses.<br><br>Not pregnant	Not pregnant General population rather than high-risk groups.<br><br>Newly married or engaged	Already have a child		Pregnancy (in this study women getting pregnant exit the trial).<br>Engaging in positive health behaviors before conception.	

		Newly married or engaged (explicitly justified by considering religion of country.)					
Stephenson et al. (2018)<br><br>Peer reviewed	Preconception health: health of women around time of conception. i.e. nutritional status.<br><br>The preconception period.	Up to 3 years before pregnancy.<br><br>3 months before pregnancy (common but not recommended by Stephenson et al.)<br><br>Reproductive age<br><br>Whenever a woman or couple decides they want to have a baby.<br><br>Fertile couples<br><br>Health before conception<br><br>Lifecourse model of critical periods:<br><br>Biological perspective - days/weeks before embryo development.<br><br>Individual perspective - intention to conceive (weeks/months before pregnancy).<br><br>Public health perspective - months/years taken to address long-term preconception risk factors, including adolescence.<br><br>Interpregnancy period<br><br>Differs by individual and their needs (e.g. overweight vs. folate).	An intention to become pregnant (not specifically planning) reproductive age woman.	Not planning a pregnancy but meeting all other attributes of preconception.	Reproductive age, fertile, decision that they want to have a baby.	Implicitly mention mothers and child's health.<br><br>Preconception health is a key determinant of pregnancy success and next generation health.<br><br>Unplanned (including mistimed and unwanted).<br><br>Pregnancy	
Szwajcer et al. (2008)<br><br>Peer reviewed	Preconception vs. pregnancy.	Women trying to conceive.<br><br>Women who had stopped using contraceptives in order to become pregnant.<br><br>Nulliparous women who appeared to have a future child wish.<br><br>Aged 20-40 years.		Life course perspective.		Motherhood	
Thompson (2017)<br><br>Peer reviewed	Women prior to pregnancy<br>Pre-pregnancy.<br><br>"Prepregnant" state.	All reproductive age persons (women and men).	Anyone of reproductive age before conception.	Reproductive age and women's lifespan (individual vs population focus).<br><br>Don't need to be planning a pregnancy.	Reproductive age<br>not pregnant<br><br>Abortion	Optimize outcomes for perinatal and infant health.	

				Contradictory cases: women who are physically incapable of pregnancy and women who do not wish to have children<br><br>Reproductive life planning<br><br>Fertility			
Toivonen (2017)<br><br>Peer reviewed scoping review	Individuals of reproductive age.<br><br>Focus on PC health behaviours	Males and females at any point prior to a potential pregnancy<br><br>Across the entire lifespan.<br><br>All women of reproductive age (PC period technically comprises any time point before conception).		Pregnancy planning status.<br><br>Pregnant or postpartum.<br><br>Define preconception period in terms of a finite number of months before pregnancy.		Child health<br>mother health behaviours, health and fertility.	
Van der Zee et al. (2013)<br><br>Peer reviewed	Preconception care.	All prospective parents.<br><br>Women considering pregnancy.<br><br>Men		Improve couples' informed decision making, providing information on reproductive options.<br><br>Couples with fertility problems vs. all couples with a pregnancy wish.<br><br>Age effects on fertility.<br><br>Publicity/keeping it a secret.	Pregnancy planning.<br><br>Contraceptive use.	Promote better reproductive outcomes.<br><br>Improve women's overall health.	
Vousden et al. (2017)<br><br>Peer reviewed	Before conception.			Time to pregnancy/ Conception.			
Weisman et al. (2008)<br><br>Peer reviewed	Before the first pregnanc[illegible] and between pregnancie[illegible]	Many women may not plan for pregnancy and 50% of pregnancies are unplanned.<br><br>Women aged 18-45<br><br>Reproductive capacity (no hysterectomy, tubal ligation or infertility).<br><br>Pregnancy intent (reporting considering becoming pregnant<br>at some time in the future).	Having a reproductive life plan.	Having a reproductive life plan.	Awareness of the importance of preconception health behaviours and health services - assuming then that women will be inclined to act before pregnancy.	To optimise health outcomes for women and their newborns.	Refers to Centers for Disease Control and Prevention's (CDC) "Recommendations to Improve Preconception Health and Health Care" - United Stages (CDC, 2006).

*This table was generated as part of the iterative concept development process. The data items entered into this table were used to generate discussion and reflection throughout the process.

References

Abbas WAK, Azar NG, Haddad LG, Umlauf MG. Preconception health status of Iraqi women after trade embargo. *Public Health Nursing* 2008; **25**(4): 295–303.

Agricola E, Gesualdo F, Carloni E, et al. Investigating paternal preconception risk factors for adverse pregnancy outcomes in a population of internet users. *Reproductive Health* 2016; **13**: 1–7.

Agricola E, Pandolfi E, Gonfiantini MV, et al. A cohort study of a tailored web intervention for preconception care. *BMC Medical Informatics and Decision Making* 2014; **14**(1): 33.

Ahrens KA, Silver RM, Mumford SL, et al. Complications and safety of preconception low-dose aspirin among women with prior pregnancy losses. *Obstetrics and Gynecology* 2016; **127**(4): 689–98.

Aranda N, Ribot B, Garcia E, Viteri FE, Arija V. Pre-pregnancy iron reserves, iron supplementation during pregnancy, and birth weight. *Early Human Development* 2011; **87**(12): 791–7.

Bastani F, Hashemi S, Bastani N, Haghani H. Impact of preconception health education on health locus of control and self-efficacy in women. *Eastern Mediterranean Health Journal* 2010; **16**(4): 396–401.

Bortolus R, Oprandi NC, Morassutti FR, et al. Why women do not ask for information on preconception health? A qualitative study. *BMC Pregnancy and Childbirth* 2017; **17**: 1–11.

Chason RJ, McLain AC, Sundaram R, et al. Preconception stress and the secondary sex ratio: A prospective cohort study. *Fertility and Sterility* 2012; **98**(4): 937–41.

Frayne DJ, Verbiest S, Chelmow D, et al. Health care system measures to advance preconception wellness: Consensus recommendations of the clinical workgroup of the National Preconception Health and Health Care Initiative. *Obstetrics and Gynecology* 2016; **127**(5):863–72.

Goossens J, Delbaere I, Dhaenens C, et al. Preconception-related needs of reproductive-aged women. *Midwifery* 2016; **33**: 64–72.

Hall JA, Mann S, Lewis G, Stephenson J, Morroni C. Conceptual framework for integrating 'Pregnancy Planning and Prevention' (P3). *Journal of Family Planning and Reproductive Health Care* 2016; **42**(1): 75–6.

Hemsing N, Greaves L, Poole N. Preconception health care interventions: A scoping review. *Sexual and Reproductive Healthcare* 2017; **14**(Supplement C): 24–32.

Lum KJ, Sundaram R, Buck Louis GM. Women's lifestyle behaviors while trying to become pregnant: Evidence supporting preconception Fguidance. *American Journal of Obstetrics and Gynecology* 2011; **205**(3): 203.e1–7.

M'hamdi HI, Sijpkens MK, de Beaufort I, Rosman AN, Steegers EAP. Perceptions of pregnancy preparation in women with a low to intermediate educational attainment: A qualitative study. *Midwifery* 2018; **59**: 62–7.

Nguyen PH, Lowe AE, Martorell R, et al. Rationale, design, methodology and sample characteristics for the Vietnam pre-conceptual micronutrient supplementation trial (PRECONCEPT): A randomized controlled study. *BMC Public Health* 2012; **12**(1): 898.

Ockhuijsen HDL, Gamel CJ, van den Hoogen A, Macklon NS. Integrating preconceptional care into an IVF programme. *Journal of Advanced Nursing* 2012; **68**(5): 1156–65.

Public Health England. Making the Case for Preconception Care: Planning and Preparation for Pregnancy to Improve Maternal and Child Health Outcomes. London, 2018.

Royal Australian and New Zealand College of Obstetricians and Gynaecologists. Pre-pregnancy counselling, 2017.

Sardasht FG, Shourab NJ, Jafarnejad F, Esmaily H. The frequency of risk factors associated with pregnancy among women seeking planned pregnancy. *Journal of Midwifery and Reproductive Health* 2017; **5**(3): 942–9.

Schisterman EF, Silver RM, Lesher LL, et al. Preconception low-dose aspirin and pregnancy outcomes: Eesults from the EAGeR randomised trial. *Lancet* 2014; **384**(9937): 29–36.

Silver RM, Ahrens K, Wong LF, et al. Low-dose aspirin and preterm birth: A randomized controlled trial. *Obstetrics and Gynecology* 2015; **125**(4): 876–84.

Sjaarda LA, Radin RG, Silver RM, et al. Preconception low-dose aspirin restores diminished pregnancy and live birth rates in women with low-grade inflammation: A secondary analysis of a randomized trial. *Journal of Clinical Endocrinology and Metabolism* 2017; **102**(5): 1495–504.

Skau JK, Nordin AB, Cheah JC, et al. A complex behavioural change intervention to reduce the risk of diabetes and prediabetes in the pre-conception period in Malaysia: Study protocol for a randomised controlled trial. *Trials* 2016; **17**(1): 215.

Stephenson J, Heslehurst N, Hall J, et al. Before the beginning: Nutrition and lifestyle in the preconception period and its importance for future health. *The Lancet* 2018; **391**(10132): 1830–41.

Szwajcer EM, Hiddink GJ, Maas L, Koelen MA, van Woerkum CM. Nutrition-related information-seeking behaviours of women trying to conceive and pregnant women: Evidence for the life course perspective. *Family Practice* 2008; **25 Suppl 1**: i99–104.

Thompson EL, Vázquez-Otero C, Vamos CA, Marhefka SL, Kline NS, Daley EM. Rethinking preconception care: A critical, women's health perspective. *Maternal and Child Health Journal* 2017; **21**(5): 1147–55.

Toivonen KI, Oinonen KA, Duchene KM. Preconception health behaviours: A scoping review. *Preventive Medicine* 2017; **96**: 1–15.

van der Zee B, de Beaufort ID, Steegers EAP, Denktas S. Perceptions of preconception counselling among women planning a pregnancy: A qualitative study. *Family Practice* 2013; **30**(3): 341–6.

Vousden NJ, Carter J, Seed PT, Shennan AH. What is the impact of preconception abdominal cerclage on fertility: Avidence from a randomized controlled trial. *Acta Obstetricia et Gynecologica Scandinavica* 2017; **96**(5): 543–6.

Weisman CS, Hillemeier MM, Chase GA, et al. Women's perceived control of their birth outcomes in the Central Pennsylvania Women's Health Study: Implications for the use of preconception care. *Women's Health Issues* 2008; **18**(1): 17–25.

Wise LA, Wesselink AK, Tucker KL, et al. Dietary fat intake and fecundability in 2 preconception cohort studies. *American Journal of Epidemiology* 2018; **187**(1): 60–74.

Young MF, Nguyen PH, Addo OY, et al. The relative influence of maternal nutritional status before and during pregnancy on birth outcomes in Vietnam. *European Journal of Obstetrics & Gynecology and Reproductive Biology* 2015; **194**: 223–7.
